# Enhanced Visible Light Photocatalytic Degradation of Organic Pollutants over Flower-Like Bi_2_O_2_CO_3_ Dotted with Ag@AgBr

**DOI:** 10.3390/ma9110882

**Published:** 2016-10-31

**Authors:** Shuanglong Lin, Miao Wang, Li Liu, Yinghua Liang, Wenquan Cui, Zisheng Zhang, Nan Yun

**Affiliations:** 1 School of Chemical engineering and Technology, Tianjin University, Tianjin 300072, China; linshuanglong@tju.edu.cn (S.L.); water_wangmiao@tju.edu.cn (M.W.); tschemlily@sina.com (L.L.); nyun@uottawa.ca (N.Y.); 2 Hebei Key Laboratory for Environment Photocatalytic and Electrocatalytic Materials, College of Chemical Engineering, North China University of Science and Technology, Tangshan 063009, China; tswqcui@gmail.com; 3 Department of Chemical and Biological Engineering, University of Ottawa, Ottawa, ON K1N 6N5, Canada

**Keywords:** semiconductors, chemical synthesis, X-ray diffraction, optical properties, catalytic properties

## Abstract

A facile and feasible oil-in-water self-assembly approach was developed to synthesize flower-like Ag@AgBr/Bi_2_O_2_CO_3_ micro-composites. The photocatalytic activities of the samples were evaluated through methylene blue degradation under visible light irradiation. Compared to Bi_2_O_2_CO_3_, flower-like Ag@AgBr/Bi_2_O_2_CO_3_ micro-composites show enhanced photocatalytic activities. In addition, results indicate that both the physicochemical properties and associated photocatalytic activities of Ag@AgBr/Bi_2_O_2_CO_3_ composites are shown to be dependent on the loading quantity of Ag@AgBr. The highest photocatalytic performance was achieved at 7 wt % Ag@AgBr, degrading 95.18% methylene blue (MB) after 20 min of irradiation, which is over 1.52 and 3.56 times more efficient than that of pure Ag@AgBr and pure Bi_2_O_2_CO_3_, respectively. Bisphenol A (BPA) was also degraded to further demonstrate the degradation ability of Ag@AgBr/Bi_2_O_2_CO_3_. A photocatalytic mechanism for the degradation of organic compounds over Ag@AgBr/Bi_2_O_2_CO_3_ was proposed. Results from this study illustrate an entirely new approach to fabricate semiconductor composites containing Ag@AgX/bismuth (X = a halogen).

## 1. Introduction

Photo-catalysts are expected to play an increasingly important role in solving some of the most challenging problems of modern society, namely energy shortages and environmental pollution [1]. However, the narrow excitation wavelength, the fast recombination rate of photogenerated electron–hole pairs and a generally poor adsorption capacity greatly inhibit the practical application of photocatalysts [2]. The search for highly active, semiconductor-based photocatalysts has received considerable attention for the applications of energy conversion and environment considerations [3,4,5,6,7]. Heterogeneous photocatalysts have been considered to be effective candidates for the conversion of solar energy to other forms; however, they also bring with them some problematic issues including their low rates of energy conversion and high recombination rates of electrons and holes [8,9]. In order to design and optimize efficient photocatalysts irradiated by solar light (λ ≥ 420 nm), which covers the largest proportion of the solar spectrum, international efforts have been expanded towards the development of new visible light-driven photocatalysts.

As a typical anionic group-containing Bi-based photocatalyst, bismuth subcarbonate (Bi_2_O_2_CO_3_) has been found to display a promising photocatalytic activity in the degradation of organic pollutants [10,11]. Bi_2_O_2_CO_3_ is typically found in a Sillén phase, in which (Bi_2_O_2_)^2+^ and (CO_3_)^2−^ layers are intergrown with the plane of the (CO_3_)^2−^ group positioned orthogonal to the plane of the (Bi_2_O_2_)^2+^ layers [12]. Although the large internal electric field and asymmetrical polarization effect may enhance the photocatalytic properties of Bi_2_O_2_CO_3_ [13,14,15], its application to photocatalytic degradation is limited by its large band gap (~3.3 eV). Thus, intensive research has been carried out on its morphological modulation [16,17], fabrication of heterojunctions [18,19,20,21,22,23,24,25,26,27], noble metal deposition [28], and elemental doping [29,30,31].

Over the past few decades, visible light plasmonic photocatalysts have aroused a significant amount of attention primarily due to their strong absorption of visible light and the ability to efficiently separate photogenerated electrons and holes due to surface plasmon resonance (SPR). In general, a plasmonic photocatalyst is a composite composed of noble metal (i.e., Ag, Au, and Pt) nanoparticles (NPs) and a polar semiconductor, such as AgX@Ag (X = Br [32], Cl [33,34], etc.). Therefore, recently, much more effort has been devoted to design Ag/AgX composite materials due to the surface plasmon resonance (SPR) of metallic Ag [35,36,37], which can dramatically enhance the absorption of visible light (VL) and provide new opportunities to develop visible-light-driven (VLD) photocatalysts [38].

Despite these advantages, photoinduced charge transfer behavior limits the promotion of photoconversion efficiency, and the reusability of Ag/AgX is not very high [39,40]. Therefore, loading Ag/AgX onto a suitable supporting material may provide an ideal solution to overcome the aforementioned drawbacks that exist for this type of photocatalyst. For instance, compared to pure Ag/AgX, the fabricated plasmon-induced Ag/AgX loaded onto graphene oxide has a significantly enhanced photocatalytic activity and stability [41].

In the present work, Ag@AgBr/Bi_2_O_2_CO_3_ composites were fabricated at room temperature by a facile oil-in-water self-assembly method and controlling the quantity of Ag@AgBr. The as-synthesized samples were characterized by X-ray powder diffraction (XRD), scanning electron microscope (SEM), BET surface area, and UV-vis diffuse reflection spectra (DRS). The photocatalytic activities were evaluated by the photocatalytic degradation of MB under simulated sunlight irradiation. Finally, the photocatalytic mechanism and effective separation of photo-induced electron–hole pairs for Ag@AgBr/Bi_2_O_2_CO_3_ composites have been investigated and discussed in detail on the basis of the experimental and computational methods. The impressive results and experimental phenomena greatly aroused our interests. It is expected that these results should contribute significantly to the development of practical applications for similar Bi-based photocatalytic materials.

## 2. Experimental

### 2.1. Photocatalyst Synthesis

All chemical reagents purchased were of analytical grade and used without further purification. The flower-like Bi_2_O_2_CO_3_ precursor was synthesized by a hydrothermal method. In a typical procedure to prepare the flower-like Bi_2_O_2_CO_3_ precursor, 3 mmol of Bi(NO_3_)_3_·5H_2_O were dissolved in 20 mL of 1 M HNO_3_, and then 2 mmol of citric acid were introduced to the solution. After 10 min of magnetic stirring, the pH of the solution was adjusted to 4–4.2 by addition of NaOH solution under vigorous stirring. The white precursor that formed was transferred to a Teflon-lined stainless steel autoclave and maintained at 160 °C for 24 h. After cooling the hydrothermal system to room temperature, the flower-like Bi_2_O_2_CO_3_ precursor was separated by centrifugation, washed several times with distilled water and ethanol, and then dried under vacuum at 80 °C for 8 h.

The Ag@AgBr/Bi_2_O_2_CO_3_ composite was prepared by a novel oil-in-water self-assembly method in the absence of light. Typically (Ag@AgBr(7 wt %)/Bi_2_O_2_CO_3_), 0.5 g Bi_2_O_2_CO_3_ powder and 0.03 g AgNO_3_ were dissolved into 40 mL deionized water and 20 mL of cetyltrimethyl ammonium bromide (CTAB 0.08 g)/carbon tetrachloride solution (nAgNO_3_:nCTAB = 1:1.2) was added dropwise at room temperature under vigorous magnetic stirring over 20 min. After the CTAB addition, the reaction mixture was magnetically stirred for another 20 min. The resulting suspension was then filtered and washed with deionized water and ethanol, respectively. The resultant AgBr/Bi_2_O_2_CO_3_ powder was dispersed in distilled water and 30 min irradiated with a 250-W metal halide lamp (Philips) equipped with wavelength cutoff filters for λ > 420 nm. The resulting sample (Ag@AgBr/Bi_2_O_2_CO_3_) was washed with distilled water and then anhydrous ethanol to remove the surfactant, and the final product (hereafter designated as Ag@AgBr/Bi_2_O_2_CO_3_) was dried at 80 °C for 8 h in the absence of light. Ag@AgBr/Bi_2_O_2_CO_3_ composites with different molar ratios of Ag@AgBr to Bi_2_O_2_CO_3_ were prepared according to the procedure described above. Pure Ag@AgBr was synthesized similarly using an oil-in-water self-assembly method with AgNO_3_ and CTAB.

### 2.2. Photocatalyst Characterization

The crystal structures and phase data for the prepared samples were determined by X-ray diffractometry (XRD) using a Rigaku D/MAX2500 PC diffractometer (Tokyo, Japan) with CuKα radiation, using an operating voltage of 40 kV and an operating current of 100 mA. The morphologies of the samples were investigated with a scanning electron microscope (SEM) (Hitachi, Chiyoda, Japan, s-4800) and by energy dispersive X-ray spectroscopy (EDX), as well as by transmission electron microscopy (TEM) (JEM-2010, JEOL Ltd., Akishima, Japan). UV-visible light (UV-vis) diffuse reflectance spectra were recorded on a UV-vis spectrometer (UV-1901, Puxi, Beijing, China). Surface areas of the samples were determined by the Brunauer–Emmett–Teller (BET) method based on the adsorption and desorption isotherms of N_2_ collected on a Quantachrome Nova 4200e automatic analyzer (Monorosb, Quantachrome, Boynton Beach, FL, USA). The photoluminescence of the powdered samples was measured with a spectrofluorometer (Hitachi, f7000). Electrochemical and photoelectrochemical measurements were performed in a constructed three-electrode quartz cell system. A Pt sheet was used as a counter electrode and Hg/Hg_2_Cl_2_/sat. KCl was used as a reference electrode, while the thin film on indium-tin oxide (ITO) was used as the working electrode for investigation. The photoelectrochemical experimental results were recorded with a CHI 660B electrochemical system (Chenhua, Shanghai, China).

### 2.3. Photocatalytic Activity

The photocatalytic activities of Ag@AgBr/Bi_2_O_2_CO_3_ samples under visible light were evaluated by the photocatalytic degradation of MB in a Pyrex glass reactor with thermostatic water outside, using a 250 W metal halide lamp (Royal Philips, Amsterdam, The Netherlands) as light source with a UV filter (λ > 400 nm, transmittance > 90%) at a distance of 10 cm from the reactor. The average visible light intensity was 125 mW·cm^−2^. Cooling was provided by an external cooling jacket, and the temperature of the reaction was controlled to 25 ± 2 °C. For each test, 0.5 g of catalyst powder was added to 100 mL of 10 mg/L MB solution [42]. Prior to irradiation, the test solution was stirred in the absence of light for 30 min. During irradiation, a 3 mL aliquot of the reaction suspension was withdrawn every 5 min and centrifuged at 10,000 rpm for 6 min to separate the particles. The collected supernatant solutions were then analyzed by a UV-vis spectrophotometer (wavelength: 400 nm < λ < 800 nm, UV-1901, Puxi, Beijing, China).

The degradation efficiency (%) was calculated as follows:

(1) $$Degradation(\%)=\frac{C_{0}-C}{C_{0}}\times \mathrm{100}\%$$

where *C*_0_ is the initial concentration of MB, and *C* is the concentration of MB at time t. Photocatalytic activities while degrading MB in the absence of light in the presence of a photocatalyst as well as under visible light irradiation in the absence of a photocatalyst were also evaluated.

The photocatalytic activities of Ag@AgBr/Bi_2_O_2_CO_3_ samples under visible light were also evaluated by the photocatalytic degradation of BPA in a Pyrex glass reactor with thermostatic water outside, using a 250 W metal halide lamp (Royal Philips, Amsterdam, The Netherlands) as light source with a UV filter (λ > 400 nm, transmittance > 90%) at a distance of 10 cm from the reactor. The average visible light intensity was 0.52 mW·cm^−2^. Cooling was provided by an external cooling jacket, and the temperature of the reaction was controlled to 25 ± 2 °C. For each test, 0.1 g of catalyst powder was added to 100 mL of 5 mg/L BPA solution. Prior to irradiation, the test solution was stirred in the absence of light for 30 min. During irradiation, a 3 mL aliquot of the reaction suspension was withdrawn every 5 min and centrifuged at 10,000 rpm for 6 min to separate the particles. The collected supernatant solutions were then analyzed by a UV-vis spectrophotometer (wavelength: 240 nm < λ < 350 nm).

### 2.4. Preparation of the Ag@AgBr/Bi_2_O_2_CO_3_ Film Electrode

The Ag@AgBr/Bi_2_O_2_CO_3_ films were prepared on indium–tin oxide (ITO) glass using a dip-coating method (Dip Coater, SYDC-100, Changtuo, Beijing, China). First, 100 mg of the Ag@AgBr/Bi_2_O_2_CO_3_ powders were dispersed in 100 mL of water, and then, treated with ultrasound for 3 h. The ITO glass was immersed in the Ag@AgBr/Bi_2_O_2_CO_3_ dispersion, and then, dip coated according to the following process: lifting height: 35 mm, dipping-pulling rate: 50 µm/s, resident time: 30 s, immerse time: 60 s, number of repeated dipping steps: 3 times. The films were dried for 30 min at 80 °C after each dipping. The dispersions were sonicated for 30 min before dipping.

## 3. Results and Discussion

### 3.1. Catalyst Characterization

**Figure 1:**
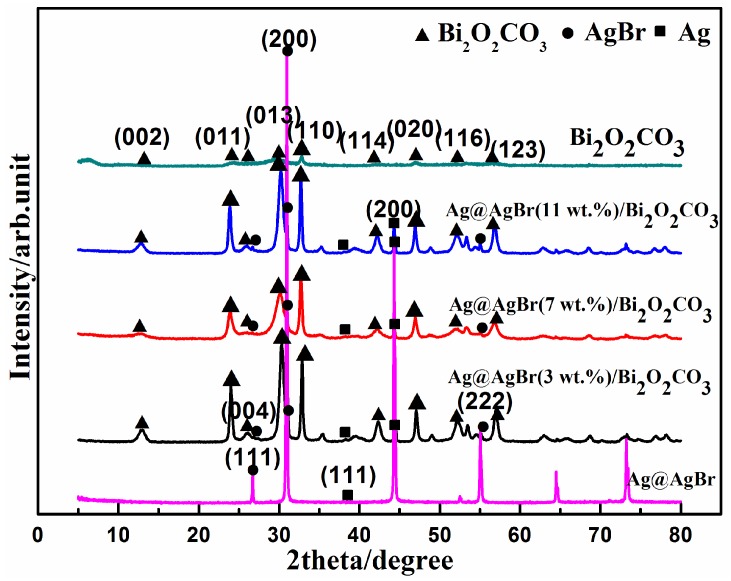
XRD patterns of the as-synthesized pure Bi_2_O_2_CO_3_, Ag@AgBr and Ag@AgBr/Bi_2_O_2_CO_3_ photocatalysts: 3 wt %, 7 wt %, and 11 wt % Ag@AgBr/Bi_2_O_2_CO_3_ composites.

The crystalline structures of the as-prepared samples were examined by X-ray diffraction. Figure 1 shows the typical XRD patterns of pure Ag@AgBr and Bi_2_O_2_CO_3_ as well as a series of Ag@AgBr/Bi_2_O_2_CO_3_ composites with various quantities of Ag@AgBr. The XRD peaks of pure Bi_2_O_2_CO_3_ agreed well with tetragonal Bi_2_O_2_CO_3_ (JCPDS card No. 41-1488; lattice constants a = 3.89 Å and c = 7.37 Å), indicating that the obtained samples had high purities. The main diffraction peak positions of products appear at 12.9°, 23.9°, 26.0°, 30.3°, 32.7°, 42.3°, 47.0°, 52.2° and 56.9°, which correspond to the (002), (011), (004), (013), (110), (114), (020), (116) and (123) crystal faces of Bi_2_O_2_CO_3_, respectively. The XRD patterns of Ag@AgBr/Bi_2_O_2_CO_3_ catalysts are essentially the same as those of Bi_2_O_2_CO_3_. The Ag@AgBr was found to be mainly composed of a AgBr phase. The XRD peaks of AgBr agreed well with the cubic crystalline phase of AgBr (JCPDS 06-0438), revealing three distinct diffraction peaks at 26.73°, 30.96° and 55.04°, which can be indexed to the (111), (200) and (222) diffraction planes of AgBr respectively, while the peaks at 38.12° (111) and 44.28° (200) can be attributed to the small quantity of elemental Ag (JCPDS 04-0783) which formed. However, the characteristic diffraction peaks corresponding to Ag@AgBr were not obvious even at high loading quantities of Ag@AgBr, indicating that the modification with Ag@AgBr did not influence the lattice structure of Bi_2_O_2_CO_3_. This may be due to the relatively small percentage contents, low diffraction intensity and high dispersion of Ag@AgBr in Bi_2_O_2_CO_3_ photocatalysts [43]. The presence of Ag@AgBr in the Ag@AgBr/Bi_2_O_2_CO_3_ samples can be confirmed by EDX analysis, as is discussed later.

**Figure 2:**
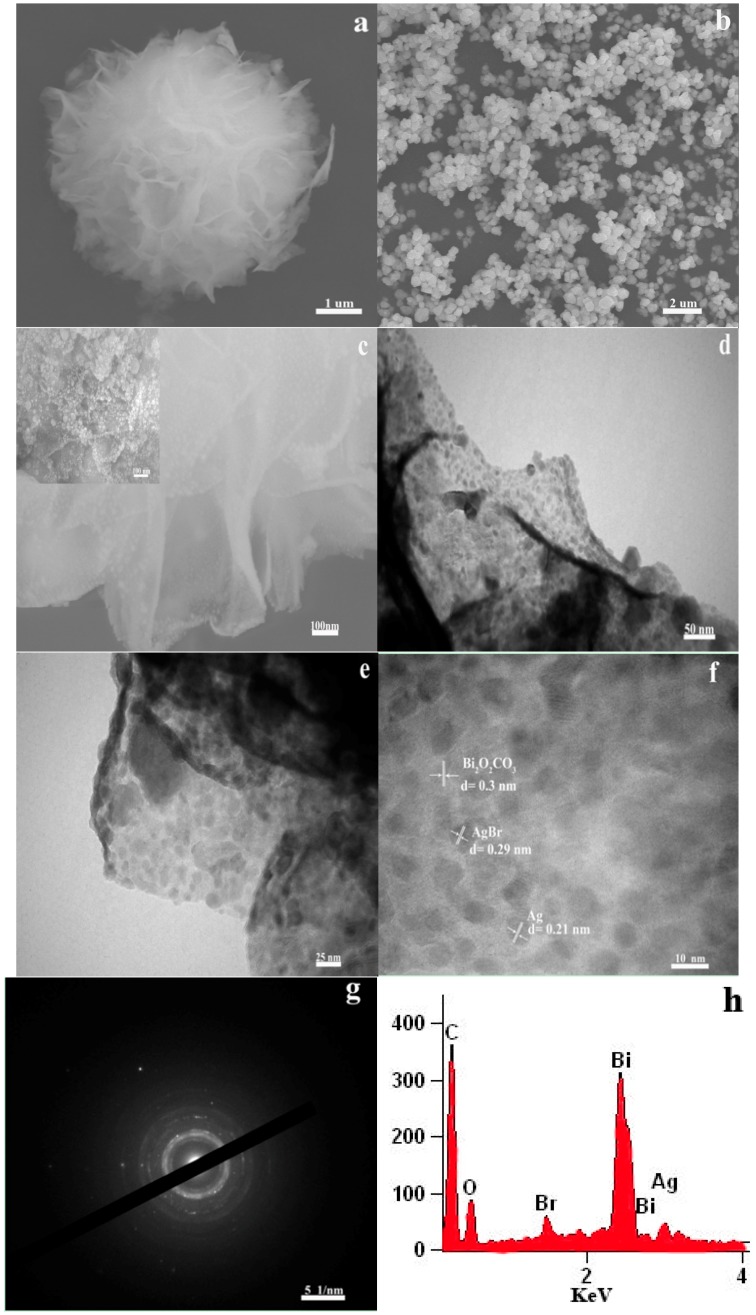
SEM images of: (**a**) Bi_2_O_2_CO_3_; (**b**) Ag@AgBr; and (**c**) Ag@AgBr(7 wt %)/Bi_2_O_2_CO_3_. The inset of (**c**): Ag@AgBr(11 wt %)/Bi_2_O_2_CO_3_. TEM image of: (**d**,**e**) Ag@AgBr(7 wt %)/Bi_2_O_2_CO_3_. HRTEM image of (**f**) Ag@AgBr(7 wt %)/Bi_2_O_2_CO_3_. SAED image of the (**g**) Ag@AgBr(7 wt %)/Bi_2_O_2_CO_3_. EDX image of (**h**) Ag@AgBr(7 wt %)/Bi_2_O_2_CO_3_.

The sizes and morphologies of the as-prepared products were further investigated by SEM. As shown in Figure 2a, the SEM image of Bi_2_O_2_CO_3_ reveals that the sample consisted of hierarchical microspheres, which are flower-like in morphology. Moreover, these flower-like microspheres are composed of smooth, regular two-dimensional (2D) nanosheets 5–15 nm in thickness with an average width of 1.5–2.0 μm. It has been reported that the internal layered structure of aurivillius phase Bi_2_O_2_CO_3_ could guide the lower growth rate along the (001) axis more so than along other axes, and thus form two-dimensional morphologies with sheet-like/plate-like structures [18]. The as-synthesized Ag@AgBr nanoparticles are nearly spherical in shape with a diameter of 0.2–0.5 μm (Figure 2b). As seen from Figure 2c, the morphology of Ag@AgBr/Bi_2_O_2_CO_3_ composites is similar to that of pure Bi_2_O_2_CO_3_, just with the deposition of many small Ag@AgBr nanoparticles (10–20 nm) on the surface, which are expected to offer an abundance of adsorption sites and to enhance photocatalytic activity. From the inset of Figure 2c, the particle size of Ag@AgBr(11 wt %)/Bi_2_O_2_CO_3_ was seen to be around 30–100 nm, and the aggregation of Ag@AgBr was observed to be significant compared to Ag@AgBr(7 wt %)/Bi_2_O_2_CO_3_. The microstructure of the Ag@AgBr/Bi_2_O_2_CO_3_ composites is further characterized by TEM and high resolution transmission electron microscopy (HRTEM). Figure 2d,e presents typical TEM images of the resulting Ag@AgBr/Bi_2_O_2_CO_3_ composites, in which the flower-like structure of Bi_2_O_2_CO_3_ serves as a novel support for Ag@AgBr nanoparticles. It was observed that many Ag@AgBr nanoparticles were deposited onto the surface of nanoplates from the flower-like Bi_2_O_2_CO_3_, and their distribution on these nanoplates is uniform. The high-resolution TEM image in Figure 2f illustrates in detail the structure of the nanojunction of Ag@AgBr/Bi_2_O_2_CO_3_ composites, in which the lattice fringes with a spacing of approximately 0.3, 0.29, and 0.21 nm can be indexed to the (013), (200), and (200) facets of Bi_2_O_2_CO_3_, AgBr and Ag, respectively. The above results further indicate the formation of heterojunctions between Bi_2_O_2_CO_3_ and Ag@AgBr. Obviously, very close contact between Bi_2_O_2_CO_3_ and Ag@AgBr components achieved by the oil-in-water self-assembly process is believed to favor the vectorial transfer of photogenerated electrons from Ag@AgBr to Bi_2_O_2_CO_3_, thus enhancing the charge separation and photocatalytic efficiency of the photocatalyst. The SAED pattern collected from the Ag@AgBr/Bi_2_O_2_CO_3_ composite is shown in Figure 2g, where polycrystalline diffraction rings can be observed. In addition, the constituents of Ag@AgBr/Bi_2_O_2_CO_3_ have been studied under the EDX spectrum method, as shown in Figure 2h. It is evident that the obtained composite is elementally composed of Ag, Br, Bi, C, and O. The EDX results, therefore, demonstrate the existence of Ag@AgBr and Bi_2_O_2_CO_3_ in the Ag@AgBr/Bi_2_O_2_CO_3_ samples. They also demonstrate that Ag@AgBr existed on the surface of Bi_2_O_2_CO_3_. Additionally, compared to pure Ag@AgBr, the Ag@AgBr nanoparticles on the surface of Bi_2_O_2_CO_3_ show uniform morphology and particle size distributions. The BET results revealed that the specific surface area and average pore size of the Ag@AgBr/Bi_2_O_2_CO_3_ nanocomposite was larger than that of pure Bi_2_O_2_CO_3_ (as shown in Table 1). The higher surface area represents a good sorption ability of such materials. In addition, the larger surface areas and bigger pore size were more favorable to the adsorption capacity of pollutants, and the porous structures could offer efficient transport pathways to reactants and more active sites, which are expected to be useful in the photocatalytic reaction. This evidence supports the enhancement of the photocatalytic activity.

**Figure 3:**
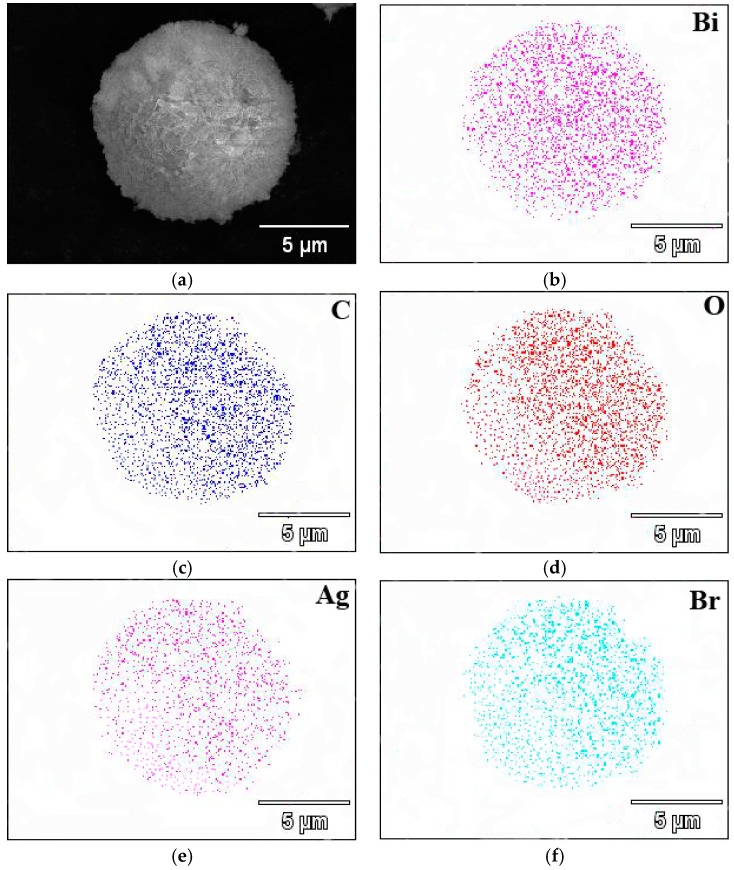
SEM image (**a**) and the corresponding elemental mapping images (**b**–**f**) of Ag@AgBr(7 wt %)/Bi_2_O_2_CO_3_.

Figure 3 displays the SEM image of the as-prepared Ag@AgBr/Bi_2_O_2_CO_3_ sample and its corresponding elemental mapping images. As can be seen, the elements Bi, C, O, Ag and Br are uniformly distributed in the spherical structure of the Ag@AgBr/Bi_2_O_2_CO_3_ composite photocatalyst nanoparticle. The results of elemental mapping in Figure 3 are evidence that Bi, C and O are from Bi_2_O_2_CO_3_, and the Br and Ag elements are evenly distributed on the obtained Ag@AgBr/Bi_2_O_2_CO_3_ [19]. This serves as solid evidence for the formation of Ag@AgBr/Bi_2_O_2_CO_3_ heterostructures. Besides, the associated elemental mapping images were obtained to evaluate the chemical uniformity within individual particles, which clearly confirmed that the Ag@AgBr have been successfully grown on the surface of the Bi_2_O_2_CO_3_ microsphere.

**Figure 4:**
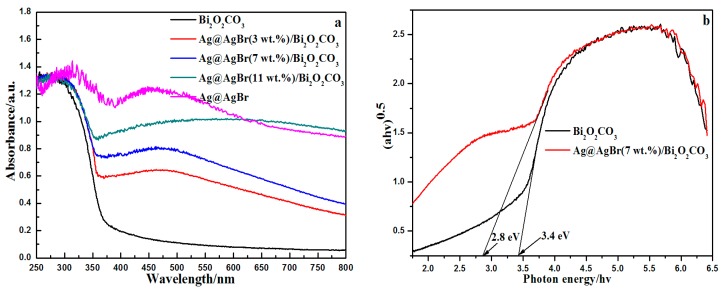
(**a**) UV-vis absorption spectra of the samples; and (**b**) (αhν)^1/2^ vs. photon energy (hν) curves of Bi_2_O_2_CO_3_ and Ag@AgBr(7 wt %)/Bi_2_O_2_CO_3_.

UV-vis diffuse reflection spectra (DRS) were acquired to determine the optical properties of the samples. Figure 4a displays the DRS of pure Ag@AgBr, pure Bi_2_O_2_CO_3_ and Ag@AgBr/Bi_2_O_2_CO_3_ samples. The pure Bi_2_O_2_CO_3_ sample boasted a weak visible light response with an absorption band edge of approximately 365 nm, while the pure Ag@AgBr showed a prominent visible light absorption, most likely due to the surface plasmon resonance (SPR) of Ag nanoparticles. Moreover, it can be seen that Ag@AgBr/Bi_2_O_2_CO_3_ samples exhibit clear visible light absorption, which can also be attributed to surface plasmon resonance, further confirming the presence of Ag nanoparticles. A red shift of the absorption edge for Ag@AgBr/Bi_2_O_2_CO_3_ was also detected. The absorption curves of the Ag@AgBr/Bi_2_O_2_CO_3_ samples exhibit distinctly enhanced visible light absorption with increasing Ag@AgBr content, suggesting that the as-prepared samples should perform with a high photocatalytic potential in the visible light region. The band gap energy of Bi_2_O_2_CO_3_ can be estimated from a plot of (αhν)^1/2^ against photon energy (hν). The intercept of the tangent with the x-axis provides a good approximation of the band gap energy for the samples. As shown in Figure 4b, the estimated band gap energy of pure Bi_2_O_2_CO_3_ was approximately 3.4 eV, and the band gap of Ag@AgBr(7 wt %)/Bi_2_O_2_CO_3_ was about 2.8 eV. The results show that, after Ag@AgBr loading, the catalyst can greatly broaden the range and improve the intensity of the visible light absorption, resulting in improved photoactivity.

**Figure 5:**
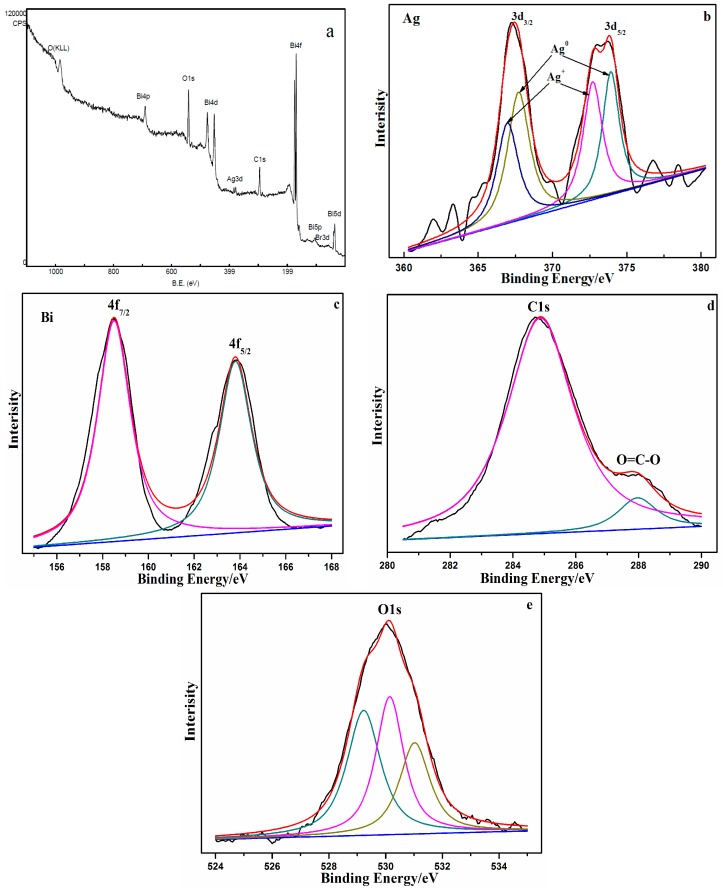
(**a**) XPS survey spectrum of Ag@AgBr(7 wt %)/Bi_2_O_2_CO_3_. High resolution XPS spectra of: (**b**) Ag 3d spectra; (**c**) Bi 4f spectra; (**d**) C 1s spectra; and (**e**) O 1s spectra.

XPS was conducted to investigate the surface chemical compositions of the Ag@AgBr/Bi_2_O_2_CO_3_ samples (Figure 5). Figure 5a gives us the typical survey spectrum of the as-obtained samples, showing that the sample consists solely of Bi, O, C, Ag and Br. In addition, the high resolution core spectrum for Ag 3d is shown in Figure 5b. The two strong peaks located at approximately 373.51 and 367.23 eV correspond to 3d_3/2_ and 3d_5/2_, respectively. The 3d_3/2_ spectrum could be fitted by two peaks located at binding energies of 373.92 and 372.71 eV, while the Ag 3d_5/2_ peak can also be divided into two separate peaks located at 367.71 and 366.97 eV. The peaks at 366.97 and 372.71 eV may be attributed to Ag^+^ from AgBr, while the peaks at 373.92 and 367.71 eV may both be assigned to metallic Ag [44]. The results of XPS analysis confirm the presence of Ag and AgBr in the prepared composite samples. Figure 5c shows the high-resolution XPS spectra of Bi 4f, the binding energies of Bi 4f_5/2_ and Bi 4f_7/2_ are 163.8 eV and 158.5 eV, indicating the existence of Bi (III). The peaks of C 1s at 284.9 eV and 288.0 eV match well with C 1s and O=C–O in Bi_2_O_2_CO_3_ (Figure 5d). The photoelectron peak for the O 1s was apparent at a binding energy E_b_ of 530.2 eV for the synthesized materials (Figure 5e). It was resolved into three peaks at 531.0 eV (adsorbed oxygen), 530.1 eV (hydroxyl oxygen) and 529.2 eV (lattice oxygen) of O 1s in Ag@AgBr/Bi_2_O_2_CO_3_. The existence of adsorbed oxygen demonstrated that O_2_ molecules deposited on the surface of Ag@AgBr/Bi_2_O_2_CO_3_. While the existence of hydroxyl oxygen demonstrated that there was a small amount of H_2_O, this is because Ag@AgBr/Bi_2_O_2_CO_3_ easily adsorbed water vapor in air. In addition, the lattice oxygen peak was thought to exist due to the Oxygen structure.

**Figure 6:**
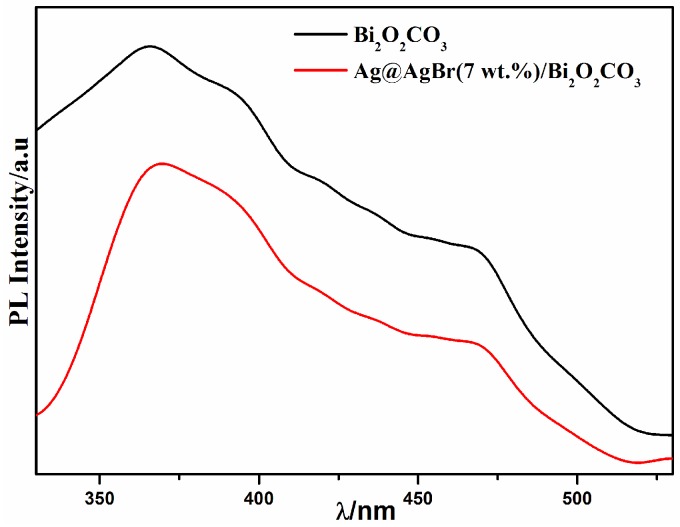
Photoluminescence (PL) spectra of pure Bi_2_O_2_CO_3_ and Ag@AgBr(7 wt %)/Bi_2_O_2_CO_3_ sample (excitation at 280 nm at room temperature).

The efficiency of charge trapping and recombination of photoinduced electron–hole pairs in the semiconductor can be also emphasized, as verified by the photoluminescence (PL) spectrum, which is useful to evaluate the photocatalytic performance of the semiconductor materials. As shown in Figure 6, Ag@AgBr(7 wt %)/Bi_2_O_2_CO_3_ exhibits similar shapes and positions with those of pure Bi_2_O_2_CO_3_. In the range of 330–530 nm, photo-luminescence intensities decreased in the order of Bi_2_O_2_CO_3_ > Ag@AgBr(7 wt %)/Bi_2_O_2_CO_3_. Nonetheless, Ag@AgBr(7 wt %)/Bi_2_O_2_CO_3_ exhibited a weaker peak because of the weak recombination of the electron–hole pairs to enhance photon efficiency.

### 3.2. Photocatalytic Activity

**Figure 7:**
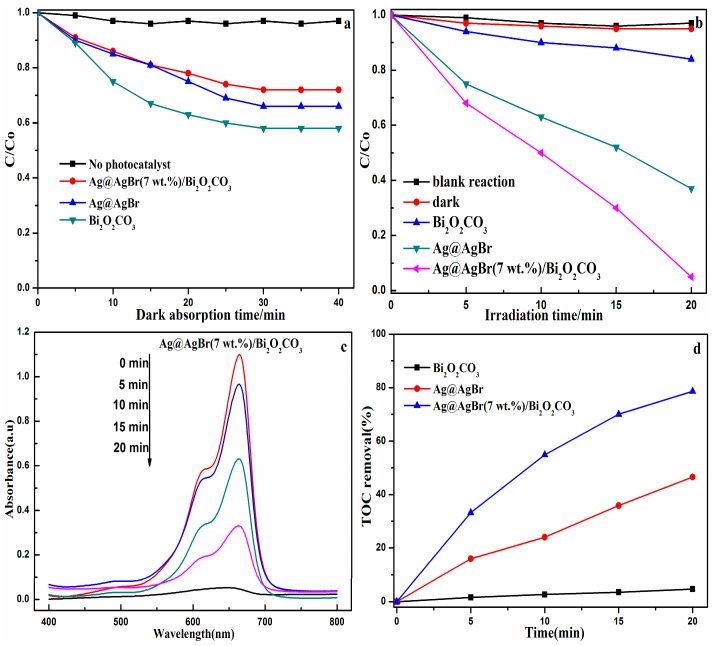
Photocatalytic adsorption (**a**); and degradation (**b**) curves of MB over the various samples. (**c**) Absorption spectra for MB solution in the presence of Ag@AgBr(7 wt %)/Bi_2_O_2_CO_3_ under visible light irradiation over time; (**d**) TOC removal of MB over various photocatalysts under visible light irradiation.

Figure 7a shows the adsorption performance of Ag@AgBr, Bi_2_O_2_CO_3_ and Ag@AgBr(7 wt %)/Bi_2_O_2_CO_3_. During the dark period, Ag@AgBr, Bi_2_O_2_CO_3_ and Ag@AgBr(7 wt %)/Bi_2_O_2_CO_3_ removed MB from the solution via adsorption. The adsorption equilibrium was reached between 25 and 30 min for these materials. Upon visible light irradiation, as shown in Figure 7b, blank reaction was also given as controls. The blank experiments, which were performed in the absence of a photocatalyst, showed no obvious changes in the concentration of MB over 20 min of reaction under visible light irradiation. Figure 7b shows the photocatalytic degradation of MB in terms of relative concentration (*C*/*C*_0_) as a function of irradiation time using Ag@AgBr(7 wt %)/Bi_2_O_2_CO_3_ nanocomposites under visible light. For comparison, the experiment was also carried out using various controls. This verifies that MB is a chemically stable organic dye that does not readily decompose without outside influence. The changes in the concentration of MB caused by the adsorption of the catalyst materials were also determined in the absence of light, yielding similar results to those that were seen in the absence of a photocatalyst. The Ag@AgBr(7 wt %)/Bi_2_O_2_CO_3_ composite displayed the highest activity under visible light irradiation, and the removal of MB reached 95.18% after 20 min, which is over 1.52 and 3.56 times more efficient than that of pure Ag@AgBr (calculated based on the equivalent Ag@AgBr content in Ag@AgBr(7 wt %)/Bi_2_O_2_CO_3_) and pure Bi_2_O_2_CO_3_, respectively). Figure 7c shows the temporal change of the UV-vis absorption spectra for solutions containing MB exposed to visible light as a function of time in the presence of Ag@AgBr(7 wt %)/Bi_2_O_2_CO_3_. It can be seen that the strong absorption peak at 664 nm decreases slightly in intensity as the irradiation time increases. Moreover, after approximately 20 min of irradiation time, the initial blue color of the MB solutions gradually faded during the process of photocatalytic degradation, which further suggests the complete destruction of the conjugated structure of MB. Figure 7d shows the total organic carbon (TOC) removal of MB as a function of reaction time. After 20 min illumination, the TOC removal by Bi_2_O_2_CO_3_, Ag@AgBr, and Ag@AgBr(7 wt %)/Bi_2_O_2_CO_3_ were 4.67%, 46.6%, and 78.64%, respectively. This further illustrated that the photocatalytic activity and mineralization ability of Ag@AgBr/Bi_2_O_2_CO_3_ are obviously superior to other investigated catalysts.

**Figure 8:**
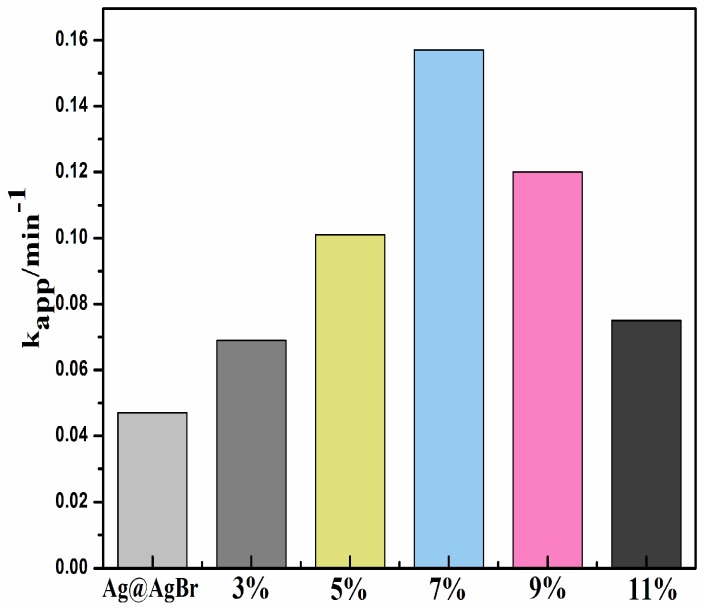
The kinetic rate constants of Ag@AgBr and Ag@AgBr/Bi_2_O_2_CO_3_ composite with different Ag@AgBr content for the photocatalytic degradation of MB under visible light irradiation.

The photocatalytic degradation of organic pollutants generally follows pseudo-first-order kinetics. As shown in Figure 8, the k_app_ values of the different samples were calculated in the following order: Ag@AgBr(7 wt %)/Bi_2_O_2_CO_3_ (0.157 min^−1^) > Ag@AgBr(9 wt %)/Bi_2_O_2_CO_3_ (0.12 min^−1^) > Ag@AgBr(5 wt %)/Bi_2_O_2_CO_3_ (0.101 min^−1^) > Ag@AgBr(11 wt %)/Bi_2_O_2_CO_3_ (0.075 min^−1^) > Ag@AgBr(3 wt %)/Bi_2_O_2_CO_3_ (0.069 min^−1^) > Ag@AgBr (0.047 min^−1^). The Ag@AgBr(7 wt %)/Bi_2_O_2_CO_3_ composite demonstrates the highest kinetic rate constant among all the samples. Its apparent rate constant is 0.157 min^−1^, which is approximately 3.34 and 2.09 times greater than those of Ag@AgBr (*k* = 0.047 min^−1^) (calculated based on the equivalent Ag@AgBr content in Ag@AgBr(7 wt %)/Bi_2_O_2_CO_3_) and Ag@AgBr(11 wt %)/Bi_2_O_2_CO_3_ (*k* = 0.075 min^−1^), respectively). In addition, the photocatalytic activity of Ag@AgBr/Bi_2_O_2_CO_3_ composites was decreased with Ag@AgBr contents exceeding 7 wt % due to the agglomeration of Ag@AgBr nanoclusters which are thought to shade the active sites on the surface of Bi_2_O_2_CO_3_ [45], as is evident based on results from the 9 wt % and 11 wt % Ag@AgBr/Bi_2_O_2_CO_3_ composites.

**Figure 9:**
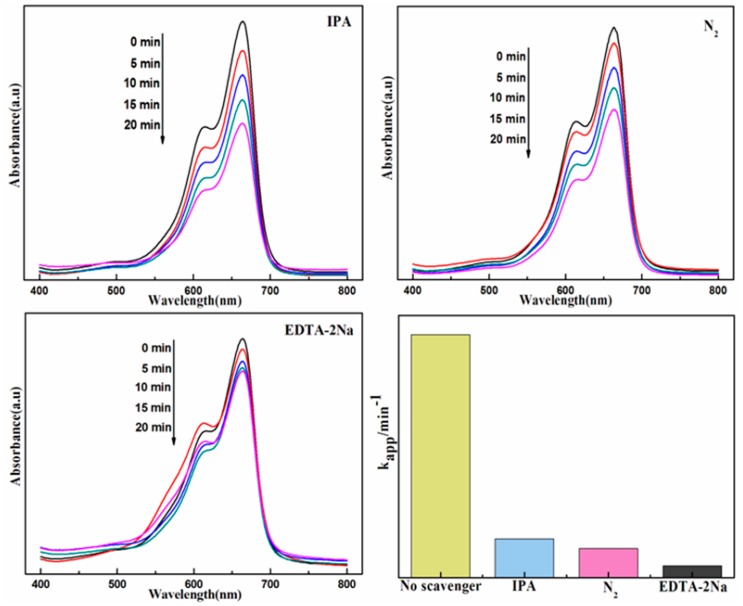
Effects of different scavengers on degradation of MB in the presence of Ag@AgBr(7 wt %)/Bi_2_O_2_CO_3_ photocatalyst under visible-light irradiation.

To investigate the primary active species involved in the photocatalytic degradation of MB in the presence of the novel Ag@AgBr/Bi_2_O_2_CO_3_ photocatalyst under visible light irradiation, sacrificial agents such as isopropyl alcohol (IPA), disodium ethylenediaminetetraacetate (EDTA-2Na) and N_2_ were used as the hydroxyl radical (·OH), hole (h^+^) and superoxide radical (·O_2_^−^) scavengers [46], respectively. The corresponding degradation kinetic constants with each scavenger species are displayed in Figure 9. When IPA was used in the degradation system, the photocatalytic degradation activity decreased significantly, indicating the presence of ·OH in solution. In addition, the degradation of MB was also significantly depressed with the addition of EDTA-2Na and N_2_, suggesting that the h^+^ and ·O_2_^−^ pathways also play a crucial role in the degradation of MB. Accordingly, the active species trapping experiments demonstrate that ·OH, h^+^ and ·O_2_^−^ are the main active species involved in the degradation of MB, which is also in agreement with the proposed photocatalytic mechanism.

**Figure 10:**
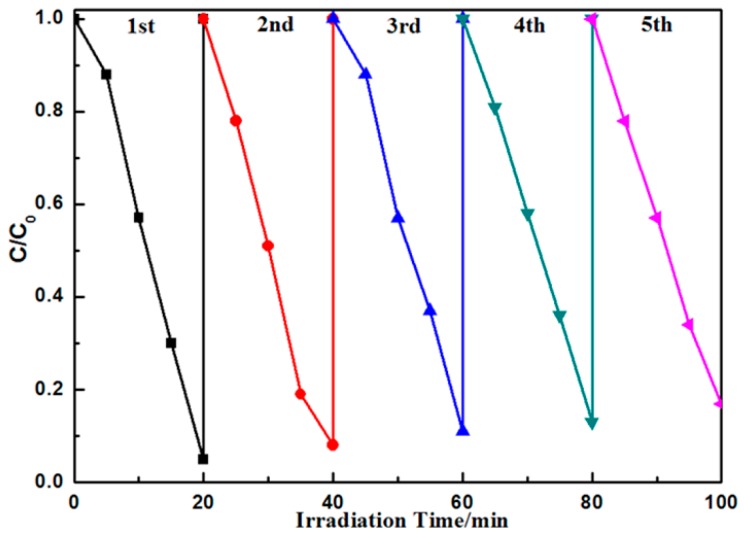
Cycling runs for the photocatalytic degradation of MB in the presence of Ag@AgBr(7 wt %)/Bi_2_O_2_CO_3_ under visible light irradiation.

Recycle experiments were carried out to evaluate the photostability of the Ag@AgBr(7 wt %)/Bi_2_O_2_CO_3_ photocatalyst under visible light irradiation. After reacting for 20 min, the photocatalyst was separated and washed several times with distilled water, after which it was dispersed into a fresh aqueous solution of MB. The change in concentration of MB during each cycle is shown in Figure 10. After five recycling iterations, Ag@AgBr(7 wt %)/Bi_2_O_2_CO_3_ did not exhibit significant loss in activity and the decomposition efficiency for MB remained above 82.6%. These results indicate that Ag@AgBr/Bi_2_O_2_CO_3_ is a stable photocatalyst during the photocatalytic oxidation of model pollutant molecules.

**Figure 11:**
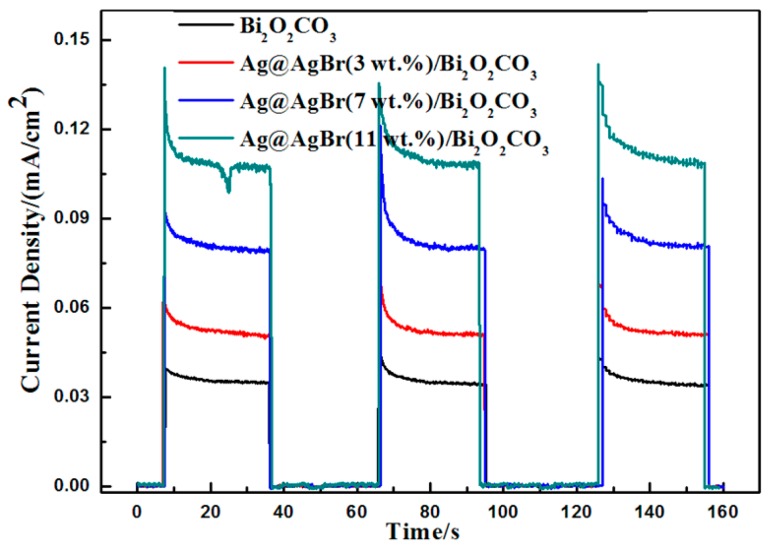
Current density-time curves of electrodes made from pure Bi_2_O_2_CO_3_, and photocatalysts of various Ag@AgBr contents.

As photocurrent responses can provide evidence for the separation rate of photogenerated electron–hole pairs in photocatalysts, transient photocurrent measurements of photocatalyst-based photoanodes were investigated. Figure 11 shows the transient curves of electrodes prepared with pure Bi_2_O_2_CO_3_, Ag@AgBr(3 wt %)/Bi_2_O_2_CO_3_, Ag@AgBr(7 wt %)/Bi_2_O_2_CO_3_ and Ag@AgBr(11 wt %)/Bi_2_O_2_CO_3_. The photocurrent responses of as-prepared samples were obtained by intermittent visible light irradiation for 30 s in 50 mL of Na_2_SO_4_ solution with a 0 V applied potential bias versus an Ag/AgCl reference electrode. Under irradiation from a Xe lamp (λ > 400 nm), both the photocurrent and dark current for Bi_2_O_2_CO_3_, Ag@AgBr(3 wt %)/Bi_2_O_2_CO_3_, Ag@AgBr(7 wt %)/Bi_2_O_2_CO_3_ and Ag@AgBr(11 wt %)/Bi_2_O_2_CO_3_ may reach their equilibrium states immediately, which is consistent with prior research [47,48]. All Ag@AgBr/Bi_2_O_2_CO_3_ composites showed higher photocurrents than that of pure Bi_2_O_2_CO_3_, which confirms that the introduction of Ag@AgBr to Bi_2_O_2_CO_3_ can promote an efficient charge transfer, indicating that Ag@AgBr/Bi_2_O_2_CO_3_ boasts an improved separation of photogenerated electron–hole pairs. However, the Ag@AgBr(7 wt %)/Bi_2_O_2_CO_3_ with the highest kinetic rate constant demonstrates lower photocurrent than that of Ag@AgBr(11 wt %)/Bi_2_O_2_CO_3_ composite. As known, photocurrent can prove the photoelectric efficiency of photocatalyst. In fact, the increase of photocatalyst performance in the heterojunctions can be more related to the degradation agent. Degradation agent reacted with holes, and oxidizing agent reacted with electronic. Degradation agent and oxidant have redox potential. When the redox potential of degradation agent was lower than that of hole, the reaction cannot happen. Relatively, when the redox potential of oxidizing agent was higher than that of electronic, the reaction also cannot respond. When one cannot respond, or the response is slow, it will lead to another one being unable to respond, or the response being slow. Therefore, there is no absolutely relationship between photocatalytic activity and photocurrent. It is possible that the composite with lower photocurrent demonstrates the highest kinetic rate constant among all the samples [49,50].

**Figure 12:**
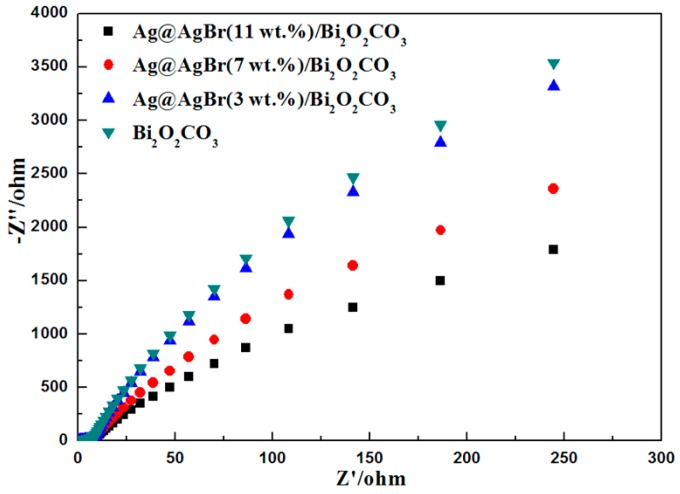
Nyquist plots of Bi_2_O_2_CO_3_, Ag@AgBr(3 wt %)/Bi_2_O_2_CO_3_, Ag@AgBr(7 wt %)/Bi_2_O_2_CO_3_ and Ag@AgBr(11 wt %)/Bi_2_O_2_CO_3_ photoelectrodes in 0.1 M Na_2_SO_4_ solution (pH = 6.7).

To validate the improved charge transfer behavior of Ag@AgBr/Bi_2_O_2_CO_3_, the EIS Nyquist plots for the photoelectrodes under illumination conditions are presented in Figure 12. The semicircular part located in the high-frequency region is associated with the charge transfer process at the photoelectrode interface and a smaller radius implies a more efficient transfer of charge [51]. The semicircle in the Nyquist plot for the Ag@AgBr/Bi_2_O_2_CO_3_ electrode exhibited a smaller radius than that of the Bi_2_O_2_CO_3_ electrode, implying that interfacial charge transfer occurred more rapidly at the interface of Ag@AgBr/Bi_2_O_2_CO_3_ than in Bi_2_O_2_CO_3_. Ag@AgBr/Bi_2_O_2_CO_3_ thus promotes an efficient separation of photogenerated electron–hole pairs.

**Figure 13:**
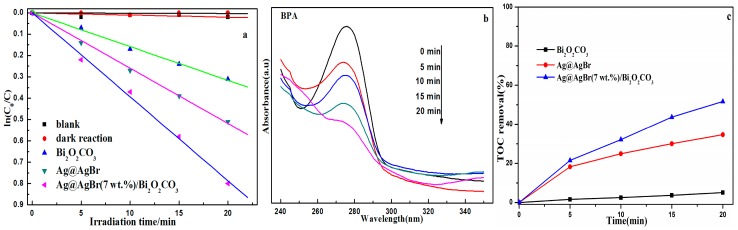
(**a**) The kinetic plots of photocatalytic degradation of BPA under visible light irradiation; (**b**) UV-vis spectra obtained at different reaction times in visible light-induced BPA photocatalytic degradation on Ag@AgBr(7 wt %)/Bi_2_O_2_CO_3_; (**c**) TOC removal of BPA over various photocatalysts under visible light irradiation

The photocatalytic activity of Ag@AgBr(7 wt %)/Bi_2_O_2_CO_3_ is assessed by studying the photodecomposition of colorless BPA in aqueous solution under visible light irradiation (λ > 420 nm). As can be seen in Figure 13a, the photolysis of BPA is negligible in the absence of a photocatalyst, suggesting that the degradation of BPA is induced by photocatalysis. The changes in the concentration of BPA caused by adsorption to the catalyst material were also determined in the absence of light, also showing a negligible degradation of BPA. Only approximately 27.1% and 40.2% BPA was shown to be degraded by pure Bi_2_O_2_CO_3_ and Ag@AgBr under visible light irradiation, respectively. The Ag@AgBr(7 wt %)/Bi_2_O_2_CO_3_ composite displayed the highest activity as well as a BPA removal efficiency of 54.9%, which was attained within 20 min. Figure 13b displays the changes in the concentration of BPA versus irradiation time. Within 20 min of visible light irradiation, the intensity of the absorption peak of BPA at 279 nm decreased dramatically as the irradiation time increased. By considering the results from photocatalytic degradation experiments, shown in Figure 13, it can be concluded that the prepared Ag@AgBr/Bi_2_O_2_CO_3_ samples exhibit better photocatalytic activities with regards to the degradation of BPA under visible light irradiation. Figure 13c shows the TOC removal of BPA as a function of reaction time. After 20 min illumination, the TOC removal by Bi_2_O_2_CO_3_, Ag@AgBr, and Ag@AgBr(7 wt %)/Bi_2_O_2_CO_3_ were 5.12%, 38.64%, and 51.68%, respectively. This further illustrated that the photocatalytic activity and mineralization ability of Ag@AgBr/Bi_2_O_2_CO_3_ are obviously superior to other investigated catalysts.

**Figure 14:**
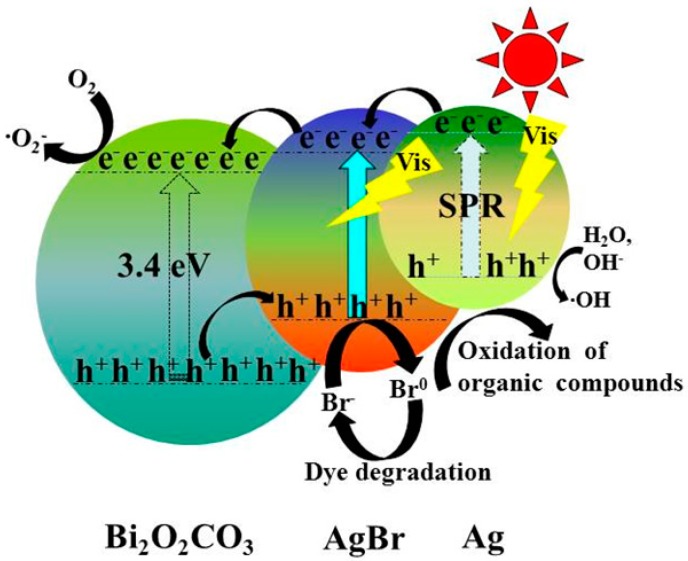
Schematic diagram of the separation of electron–hole pairs over Ag@AgBr/Bi_2_O_2_CO_3_ under visible light irradiation.

On the basis of the above experimental results and discussion, a possible mechanism describing the degradation of MB by Ag@AgBr/Bi_2_O_2_CO_3_ under visible light irradiation is illustrated in Figure 14. The band gap of Bi_2_O_2_CO_3_ is 3.4 eV and thus it cannot be excited under visible light irradiation. However, as a narrow band gap semiconductor, in AgBr (2.6 eV), there is an interband transition in electrons of the valence band (VB) and the electron–hole pairs can be segregated under visible light irradiation [52]. In addition, the surface plasmon resonance (SPR) of Ag nanoparticles in the Ag@AgBr/Bi_2_O_2_CO_3_ composite further enhances visible light absorption. The energetic electrons from the plasmon-excited Ag nanoparticles move to the conduction band of AgBr. On the basis of the relative position of the conduction band, photogenerated electrons transfer from the conduction band of AgBr (−0.6 eV) [53] to the CB of Bi_2_O_2_CO_3_ (0.16 eV) [42]. The electrons in the CB of Bi_2_O_2_CO_3_ can be trapped by dissolved O_2_ to generate reaction-active ·O_2_^−^, which may further oxidize MB. This process is beneficial for the separation of photogenerated charge carriers, leading to a higher photocatalytic activity due to the incorporation of Ag@AgBr nanoparticles.

In addition, the holes in Bi_2_O_2_CO_3_ move to the valence band of AgBr, after which the photogenerated holes on the surface of AgBr react with Br^−^ to form Br^0^. The Br^0^ atoms are reactive radical species that oxidize surface-adsorbed organic pollutants, reducing the Br^0^ atoms back to Br^−^. The resultant Br^−^ may then react with Ag^+^ to reform AgBr, maintaining the stability of the photocatalyst sample under visible light irradiation [54]. At the same time, the photogenerated holes in Ag nanoparticles are powerful oxidative species that can directly react with water (or hydroxyl) to form powerful ·OH, leading to the subsequent decomposition of MB [55,56]. Meanwhile, the photogenerated holes can directly oxidize MB to form the final products. The major reaction steps during the photocatalytic process are listed as follows:

(2) $$Ag+hv\to Ag(e^{-}+h^{+})$$

(3) $$AgBr+hv\to AgBr(e^{-}+h^{+})$$

(4) $$Ag(e^{-})+AgBr(e^{-})+Bi_{2}O_{2}CO_{3}\to Bi_{2}O_{2}CO_{3}(e^{-})+Ag+AgBr$$

(5) $$Bi_{2}O_{2}CO_{3}(e^{-})+O_{2}\to Bi_{2}O_{2}CO_{3}+\cdot O_{2}^{-}$$

(6) $$H_{2}O/OH^{-}+h^{+}\to \cdot OH$$

(7) $$\cdot OH/\cdot O_{2}^{-}+MB\to Products$$

(8) $$Br^{-}+h^{+}\to Br^{0}$$

(9) $$Br^{0}+MB\to Br^{-}+Products$$

(10) $$MB+h^{+}\to CO_{2}+H_{2}O+Other\;products$$

On the basis of the above results, this possible mechanism for the enhancement of the photocatalytic activity for Ag@AgBr/Bi_2_O_2_CO_3_ heterojunctions may be proposed. Firstly, the decoration of Ag@AgBr nanoparticles and Bi_2_O_2_CO_3_ is beneficial to the improvement of light absorbing capacity, which has been demonstrated by UV-vis DRS analysis. Secondly, a large surface area can enhance the absorption activity and increase the number of reaction sites. Thirdly, creating Ag@AgBr/Bi_2_O_2_CO_3_ heterojunctions can enhance charge transfer and inhibit the recombination of electron–hole pairs, which is implicit to improve photocatalytic activity. In summary, Ag@AgBr/Bi_2_O_2_CO_3_ can induce more electrons and holes to transfer to reaction sites and to generate more reactive species, which leads to a better photocatalytic activity than that of pure Bi_2_O_2_CO_3_ or Ag@AgBr.

## 4. Conclusions

In summary, novel visible light-driven, flower-like Ag@AgBr/Bi_2_O_2_CO_3_ composites were prepared via a simple oil-in-water self-assembly method. Subsequently, Ag@AgBr/Bi_2_O_2_CO_3_ boasts an excellent photocatalytic activity with regards to the degradation of both MB and BPA. The experimental results revealed that the photocatalytic activity of this novel Ag@AgBr/Bi_2_O_2_CO_3_ nanophotocatalyst was superior to that of pure Ag@AgBr and Bi_2_O_2_CO_3_ under visible light irradiation. Most of all, among the as-prepared samples, the Ag@AgBr/Bi_2_O_2_CO_3_ composite with 7 wt % Ag@AgBr showed the highest photocatalytic performance (95.18% MB degraded) in the removal of organic pollutants. The presence of Ag@AgBr nanoparticles evidently promoted interfacial charge transfer and thus efficiently reduced the rate of photoinduced electron–hole recombination. In addition, Ag@AgBr/Bi_2_O_2_CO_3_ has a large specific surface area, which provides a larger number of active sites for adsorption and degradation of MB dyes. The photocatalyst also demonstrated a high stability during photoreactions and no obvious deactivation was found for the recycled catalyst after five test runs. The present results suggest that Ag@AgBr/Bi_2_O_2_CO_3_ is a promising candidate with a high reactivity and stability with regards to the photocatalytic degradation of organic pollutants using solar energy. Therefore, Ag@AgX (X = Cl, Br, I)/bismuth-based semiconductor composites may have great potential in environmental as well as energy storage and conversion fields such as water treatment, green catalysis, fuel cells and carbon dioxide conversion.

## Figures and Tables

**Table 1:** Specific surface areas and average pore size of the prepared samples.

Photocatalyst	Bi_2_O_2_CO_3_	Ag@AgBr (3 wt %)/Bi_2_O_2_CO_3_	Ag@AgBr (5 wt %)/Bi_2_O_2_CO_3_	Ag@AgBr (7 wt %)/Bi_2_O_2_CO_3_	Ag@AgBr (9 wt %)/Bi_2_O_2_CO_3_	Ag@AgBr (11 wt %)/Bi_2_O_2_CO_3_
Surface area/m^2^·g^−1^	12.59	15.15	18.1	22.33	25.16	28.91
Average pore size/nm	21.15	21.43	21.64	22.02	21.75	21.25
